# Sex‐ and age‐based differences in the natural history and outcome of dilated cardiomyopathy

**DOI:** 10.1002/ejhf.1216

**Published:** 2018-06-03

**Authors:** Brian P. Halliday, Ankur Gulati, Aamir Ali, Simon Newsome, Amrit Lota, Upasana Tayal, Vassilios S. Vassiliou, Monika Arzanauskaite, Cemil Izgi, Kaushiga Krishnathasan, Arvind Singhal, Kayla Chiew, John Gregson, Michael P. Frenneaux, Stuart A. Cook, Dudley J. Pennell, Peter Collins, John G.F. Cleland, Sanjay K. Prasad

**Affiliations:** ^1^ Cardiovascular Research Centre and Cardiovascular Magnetic Resonance Unit Royal Brompton Hospital London UK; ^2^ National Heart & Lung Institute Imperial College London UK; ^3^ London School of Hygiene and Tropical Medicine London UK; ^4^ Norwich Medical School University of East Anglia Norwich UK; ^5^ National Heart Centre Singapore Singapore; ^6^ Robertson Centre for Biostatistics University of Glasgow Glasgow UK

**Keywords:** Dilated cardiomyopathy, Sex, Age, Outcome

## Abstract

**Aim:**

To evaluate the relationship between sex, age and outcome in dilated cardiomyopathy (DCM).

**Methods and results:**

We used proportional hazard modelling to examine the association between sex, age and all‐cause mortality in consecutive patients with DCM. Overall, 881 patients (290 women, median age 52 years) were followed for a median of 4.9 years. Women were more likely to present with heart failure (64.0% vs. 54.5%; P = 0.007) and had more severe symptoms (P < 0.0001) compared to men. Women had smaller left ventricular end‐diastolic volume (125 mL/m^2^ vs. 135 mL/m^2^; P < 0.001), higher left ventricular ejection fraction (40.2% vs. 37.9%; P = 0.019) and were less likely to have mid‐wall late gadolinium enhancement (23.0% vs. 38.9%; P < 0.0001). During follow‐up, 149 (16.9%) patients died, including 41 (4.7%) who died suddenly. After adjustment, all‐cause mortality [hazard ratio (HR) 0.61, 95% confidence interval (CI) 0.41–0.92; P = 0.018] was lower in women, with similar trends for cardiovascular (HR 0.60, 95% CI 0.35–1.05; P = 0.07), non‐sudden (HR 0.63, 95% CI 0.39–1.02; P = 0.06) and sudden death (HR 0.70, 95% CI 0.30–1.63; P = 0.41). All‐cause mortality (per 10 years: HR 1.36, 95% CI 1.20–1.55; P < 0.0001) and non‐sudden death (per 10 years: HR 1.51, 95% CI 1.26–1.82; P < 0.00001) increased with age. Cumulative incidence curves confirmed favourable outcomes, particularly in women and those <60 years. Increased all‐cause mortality in patients >60 years of age was driven by non‐sudden death.

**Conclusion:**

Women with DCM have better survival compared to men, which may partly be due to less severe left ventricular dysfunction and a smaller scar burden. There is increased mortality driven by non‐sudden death in patients >60 years of age that is less marked in women. Outcomes with contemporary treatment were favourable, with a low incidence of sudden death.

## Introduction

Dilated cardiomyopathy (DCM) is a heterogeneous condition manifest in a diverse group of patients due to a combination of underlying genetic susceptibility and environmental insults.^1^ The prognosis of many patients with DCM remains poor and more precise risk stratification and personalised therapy may considerably improve outcomes. Sex and age are two simple, universally available patient characteristics that deserve consideration.

Data from large registries suggest that women with heart failure (HF) have better transplant‐free survival compared to men.^2^ Whether this relates to a higher proportion of non‐ischaemic HF in women or whether this is independent of aetiology remains controversial.^3^ DCM is known to affect men more commonly than women, however detailed data comparing differences in disease phenotype, severity and outcome between sexes are lacking.^4^

The DANISH (Danish Study to Assess the Efficacy of ICDs in Patients with Non‐ischemic Systolic Heart Failure on Mortality) found that implantation of a cardioverter‐defibrillator (ICD) did not reduce overall mortality.^5^ Whilst meta‐analyses of trials have suggested mortality benefit with ICD implantation, many of the patients in these studies were not treated with contemporary HF therapy, known to reduce sudden death.^6^, ^7^ More precise selection of patients with DCM for ICD is required. Subgroup analysis of the DANISH demonstrated a mortality benefit with ICD implantation in patients aged <59 years and a trend towards worse outcomes in those >68 years. The explanation for these findings is unclear but a higher rate of death from competing causes later in life may dilute the benefit of an ICD.^8^ It is possible that malignant arrhythmia in older patients signals advanced disease and a poor prognosis from competing causes that cannot be improved by ICD implantation. Equally, it is possible that those presenting later in life have a lower incidence of ventricular arrhythmias.^9^ Examining the rates of death from non‐sudden and sudden causes according to sex and age could help inform management strategy.

## Methods

**Figure 1:**
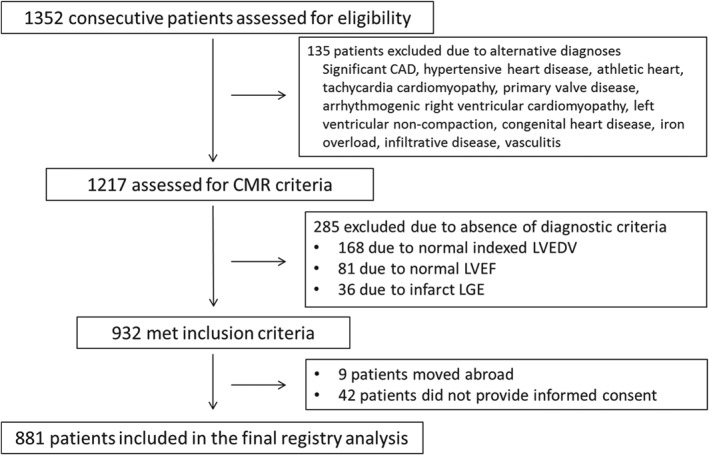
Identification, inclusion and exclusion of patients from the study cohort. CAD, coronary artery disease; CMR, cardiovascular magnetic resonance; LGE, late gadolinium enhancement; LVEDV, left ventricular end‐diastolic volume; LVEF, left ventricular ejection fraction.

Consecutive patients with suspected DCM referred to our adult cardiomyopathy service or for cardiovascular magnetic resonance (CMR) between 2000 and 2011 were screened. The study complied with the Declaration of Helsinki, was approved by the National Research Ethics Service and participants entered in the registry provided written informed consent (*Figure* 1). The final registry of 881 patients included 472 previously reported cases who underwent extended follow‐up for the purpose of this report.^10^ All patients underwent CMR at baseline using a standardized protocol for image acquisition and analysis, as previously described.^11^ The inclusion criterion was a diagnosis of DCM,^12^ based on reduced left ventricular ejection fraction (LVEF) and elevated left ventricular end‐diastolic volume indexed to body surface area (LVEDVi) compared to published age‐ and sex‐specific reference values.^13^ Exclusion criteria (*Figure* 1) included ischaemic heart disease defined as a > 50% stenosis in a major coronary artery, evidence of inducible ischaemia on functional testing, or infarct late gadolinium enhancement (LGE) patterns on CMR. In addition, ischaemic heart disease was excluded by invasive coronary angiography in 78.4%. A further 7.1% had functional imaging without evidence of inducible ischaemia. Of the remaining patients (of whom 41.1% women), none had angina, all were considered to be at low risk of coronary artery disease by their attending physicians and the majority (*n* = 82; 9.2%) were aged <40 years; accordingly, coronary angiography was not performed.^14^

Patient follow‐up was performed using postal questionnaires, telephone interview and by accessing information from general practitioners, cardiologists and hospital notes. Deaths were identified through the UK Health and Social Care Information Service. Follow‐up duration was measured from the CMR scan until last confirmed contact with the patient or the date of death. The primary endpoint was all‐cause mortality. Secondary endpoints were cardiovascular, non‐sudden and sudden cardiac death. The cause of death was confirmed by a committee of cardiologists using medical records, post‐mortem results and death certificates in line with guidance.^15^ Sudden death was defined as ‘unexpected death either within 1 h of the onset of cardiac symptoms in the absence of progressive cardiac deterioration; during sleep; or within 24 h of last being seen alive’.^15^

### Statistical analysis

Baseline characteristics were compared between men and women and those aged above or below 60 years of age using the Mann–Whitney U test for continuous data and the Fisher's exact test for categorical data. Associations between age (as a continuous variable) and sex and each endpoint were examined using univariable and multivariable proportional hazard modelling. Multivariable analyses were adjusted for important prognostic baseline covariates including age, sex, LVEF, New York Heart Association (NYHA) functional class, left bundle branch block (LBBB), atrial fibrillation (AF), smoking status and the presence or absence of mid‐wall LGE on CMR, as well as the presence or absence of an ICD or cardiac resynchronisation therapy (CRT) device as a time‐varying covariate. Cumulative incidence curves were generated for endpoints with event times measured from the baseline CMR date for up to 10 years.

## Results

The study population included 881 patients. The median age was 52 (interquartile range: 42–63) years, the median LVEF was 39% and 290 (32.9%) were women.

### Sex‐based differences in baseline characteristics and disease phenotype

Baseline characteristics are presented in Table 1. Women were less likely to have a history of AF (P < 0.0001) and alcohol excess (P < 0.0001) and more likely to have LBBB (P < 0.0001) and a history of previous chemotherapy (P < 0.0001) compared to men. In addition, 18 (6.2%) women had a presentation in the peripartum period. There was a trend towards women more frequently having a family history of DCM compared to men (P = 0.054). Three patients, one women and two men, were known to have a pathogenic or likely pathogenic mutation in LMNA. HF was more likely to be the presenting indication in women compared to men (64.1% vs. 54.5%; P = 0.007) whilst a greater proportion of men (22.0% vs. 13.8%) were referred after presenting with arrhythmia (P = 0.004). In keeping with this, NYHA class was worse in women compared to men (P < 0.0001). However, on CMR, women had smaller LVEDVi (P < 0.001), indexed right ventricular end‐diastolic volumes (P < 0.0001) and indexed left atrial volume (LAVi; P < 0.0001), higher LVEF (P = 0.019) and right ventricular ejection fraction (P < 0.0001) and a lower prevalence of mid‐wall LGE (P < 0.0001). The results remained qualitatively the same after indexing values using height rather than body surface area. Apart from a higher prescription rate of angiotensin receptor blockers in women compared to men (P = 0.04), pharmacological therapies for HF were similar between sexes (Table 2). In addition, there were no significant differences in prescribed therapies between sexes when patients with LVEF ≤40% were analysed individually.

**Table 1:** Baseline characteristics

	**Sex**			**Age**		
	**Men (n = 591)**	**Women (n = 290)**	**P‐value**	**<60 (n = 597)**	**≥60 (n = 284)**	**P‐value**
Age (years)	52 (14.8)	53 (15.1)	0.099	44 (10.8)	69 (6.1)	–
Male gender	–	–	–	418 (70.0)	173 (60.9)	0.009
Body surface area (m^2^)	2.05 (0.20)	1.77 (0.19)	<0.0001	1.97 (0.24)	1.92 (0.22)	0.006
Heart rate (b.p.m.)	72.7 (14.4)	74.0 (14.2)	0.079	73.1 (14.4)	73.1 (14.4)	0.96
SBP (mmHg)	120.2 (17.3)	120.2 (18.0)	0.89	118.3 (17.3)	124.2 (17.4)	<0.0001
DBP (mmHg)	72.9 (10.9)	71.4 (10.4)	0.041	71.9 (11.0)	73.4 (10.4)	0.072
Smoker	117 (19.8)	32 (11.0)	0.001	122 (20.4)	27 (9.5)	<0.0001
Alcohol excess	97 (16.4)	5 (1.7)	<0.0001	76 (12.7)	26 (9.2)	0.14
Atrial fibrillation/flutter	140 (23.7)	28 (10.0)	<0.0001	86 (14.4)	82 (27.9)	<0.0001
Hypertension	123 (20.8)	68 (23.4)	0.38	99 (16.6)	92 (32.4)	<0.0001
Diabetes mellitus	50 (8.5)	27 (9.3)	0.70	44 (7.4)	33 (11.6)	0.041
Hypercholesterolaemia	124 (21.0)	55 (19.0)	0.53	92 (15.4)	87 (30.6)	<0.0001
Cerebrovascular accident	8 (1.3)	3 (1.1)	1.00	6 (1.0)	5 (1.7)	0.52
Family history of DCM	50 (8.5)	37 (12.8)	0.054	73 (12.3)	14 (4.9)	<0.001
Family history of SCD	39 (6.6)	24 (8.3)	0.40	48 (8.1)	15 (5.3)	0.16
Previous chemotherapy	28 (9.7)	6 (1.0)	<0.0001	18 (3.0)	16 (5.6)	0.01
Peripartum presentation	0 (0)	18 (6.2)	<0.0001	18 (3.0)	0 (0)	<0.0001
Neuromuscular disease	7 (1.2)	1 (0.3)	0.21	8 (1.3)	0 (0)	0.044
Left bundle branch block	134 (22.7)	124 (42.9)	<0.0001	140 (23.5)	118 (41.8)	<0.0001
NYHA class			<0.0001			0.001
I	267 (45.3)	88 (30.8)		263 (44.1)	92 (32.9)	
II	231 (39.2)	125 (43.7)	219 (36.7)	137 (48.9)
III/IV	92 (15.6)	73 (25.5)	114 (19.1)	51 (18.2)
Indications						
Heart failure	322 (54.5)	186 (64.1)	0.007	346 (57.9)	162 (57.0)	0.83
Arrhythmic	130 (22.0)	40 (13.8)	0.004	116 (19.4)	54 (19.0)	0.93
Family screening	25 (4.2)	15 (3.4)	0.61	38 (6.3)	2 (0.7)	<0.0001
Other	114 (19.2)	49 (16.9)	0.41	120 (20.1)	43 (15.1)	0.08
CMR measurements						
LVEDVi^BSA^ (mL/m^2^)	135.4 (43.3)	125.3 (35.2)	<0.001	132.2 (42.1)	131.8 (39.0)	0.81
LVEDVi^height^ (mL/m)	154.9 (50.3)	135.4 (38.1)	<0.0001	149.9 (50.2)	145.6 (41.4)	0.54
LVEF (%)	37.9 (12.9)	40.2 (12.0)	0.019	39.1 (13.0)	37.5 (11.8)	0.025
LV mass index^BSA^ (g/m^2^)	100.1 (27.9)	87.9 (25.6)	<0.0001	95.6 (27.9)	97.2 (27.3)	0.33
LV mass index^height^ (g/m)	115.1 (34.2)	95.2 (28.2)	<0.0001	109.0 (35.1)	107.9 (30.6)	0.91
RVEDVi^BSA^ (mL/m^2^)	94.5 (27.0)	79.1 (21.1)	<0.0001	92.5 (26.0)	83.2 (25.7)	<0.0001
RVEDVi^height^ (mL/m)	108.2 (31.0)	86.3 (24.6)	<0.0001	105.0 (30.9)	92.8 (29.1)	<0.0001
RVEF (%)	48.9 (13.6)	55.4 (14.9)	<0.0001	50.0 (14.4)	53.3 (13.9)	0.003
LAVi^BSA^ (mL/m^2^)	68.6 (26.9)	61.0 (24.0)	<0.0001	64.1 (24.3)	70.3 (29.5)	0.001
LAVi^height^ (mL/m)	78.6 (31.1)	65.9 (25.6)	<0.0001	72.7 (28.2)	78.4 (33.3)	0.014
LGE (presence)	229 (38.9)	66 (23.0)	<0.0001	189 (31.9)	106 (37.5)	0.11

BSA, body surface area; CMR, cardiovascular magnetic resonance; DBP, diastolic blood pressure; DCM, dilated cardiomyopathy; LAVi, indexed left atrial volume; LGE, late gadolinium enhancement; LV, left ventricular; LVEDVi, indexed left ventricular end‐diastolic volume; LVEF, left ventricular ejection fraction; LVESVi, indexed left ventricular end‐systolic volume; NYHA, New York Heart Association; RVEDVi, indexed right ventricular end‐diastolic volume; RVEF, right ventricular ejection fraction; RVESVi, indexed right ventricular end‐systolic volume; SBP, systolic blood pressure; SCD, sudden cardiac death.

**Table 2:** Prescribed medical therapies at baseline based on age, sex and left ventricular ejection fraction

	**All**			**LVEF ≤ 40%**			LVEF>40%		
	**Men(n = 591)**	**Women(n = 290)**	**P‐value**	**Men(n = 322)**	**Women(n = 132)**	**P‐value**	**Men(n = 269)**	**Women(n = 158)**	**P‐value**
Beta‐blocker	433 (73.4)	209 (72.1)	0.69	266 (82.6)	100 (75.8)	0.12	167 (62.1)	109 (69.0)	0.17
ACE inhibitor	433 (73.3)	198 (68.3)	0.13	254 (78.9)	94 (71.2)	0.09	179 (66.5)	104 (65.8)	0.92
ARB	109 (18.5)	71 (24.5)	0.041	59 (18.3)	33 (25.0)	0.12	50 (18.6)	38 (24.1)	0.22
MRA	195 (33.0)	108 (37.4)	0.2	147 (45.7)	68 (51.5)	0.26	48 (36.4)	40 (25.3)	0.08
Loop diuretic	262 (44.3)	125 (43.1)	0.77	201 (62.4)	90 (68.2)	0.28	61 (22.7)	35 (22.2)	1.0
	**All**			**LVEF ≤ 40%**			**LVEF > 40%**			
	**<60(n = 597)**	**≥60(n = 284)**	**P‐value**	**<60(n = 295)**	**≥60(n = 159)**	**P‐value**	**<60(n = 302)**	**≥60(n = 125)**	**P‐value**	
Beta‐blocker	435 (72.9)	207 (73.1)	1.0	246 (83.4)	120 (75.5)	0.05	189 (62.6)	87 (69.6)	0.18	
ACE inhibitor	429 (71.9)	202 (71.1)	0.87	225 (76.3)	123 (77.4)	0.82	204 (67.5)	79 (63.2)	0.43	
ARB	113 (19.0)	67 (23.7)	0.11	63 (21.4)	29 (18.2)	0.46	50 (16.6)	38 (30.4)	0.002	
MRA	208 (34.9)	95 (33.5)	0.7	153 (51.9)	62 (39.0)	0.01	55 (18.2)	33 (26.4)	0.07	
Loop diuretic	233 (39.0)	154 (54.2)	<0.0001	182 (61.7)	109 (68.6)	0.15	51 (16.9)	45 (36.0)	0.0001	

ACE, angiotensin‐converting enzyme; ARB, angiotensin II receptor blocker; LVEF, left ventricular ejection fraction; MRA, mineralocorticoid receptor antagonist.

### Age‐based differences in baseline characteristics and disease phenotype

Patients aged ≥60 years had worse NYHA class (P = 0.001), were more likely to be prescribed loop diuretics (P = 0.0001) and had higher systolic blood pressures (P < 0.001). There was also a lower prescription of beta‐blockers (P = 0.05) and mineralocorticoid receptor antagonists (P = 0.01) to patients with a LVEF <40% who were over 60 years of age (Table 2). Those over 60 years of age were also more likely to have a history of AF (P < 0.0001), hypertension (P < 0.0001) and LBBB (P < 0.0001) but less likely to have a family history of DCM (P < 0.001) or to be referred in the context of family screening (P < 0.001). On CMR, those aged >60 years had lower LVEF (P < 0.001) and greater LAVi (P < 0.001).

### Primary and secondary endpoints

During follow‐up, 149 (16.9%) patients died, 99 (11.2%) due to cardiovascular causes (including 50 HF and 41 sudden deaths) and a further 50 (5.7%) due to non‐cardiovascular causes (including cancer, sepsis, lung disease, gastrointestinal haemorrhage, massive haemoptysis and small bowel obstruction). Rate of events per 100 patient‐years by sex and age are included in Table 3.

**Table 3:** Rate of events per 100 patient‐years by sex and age group

	**Rate per 100 patient‐years (95% CI)**			
	**Men (n = 591)**	**Women (n = 290)**	**<60 years (n = 597)**	**≥60 years (n = 284)**
All‐cause mortality	3.6 (3.0–4.3)	2.3 (1.7–3.2)	2.4 (1.9–3.0)	4.9 (3.9–6.2)
Cardiovascular	2.5 (2.0–3.1)	1.4 (0.9–2.1)	1.8 (1.4–2.3)	2.8 (2.1–3.8)
Non‐sudden death	2.6 (2.1–3.2)	1.7 (1.2–2.5)	1.5 (1.2–2.0)	4.1 (3.2–5.3)
Sudden cardiac death	1.0 (0.7–1.4)	0.6 (0.3–1.1)	0.9 (0.6–1.3)	0.8 (0.5–1.5)

CI, confidence interval.

### Association between sex and outcome

**Figure 2:**
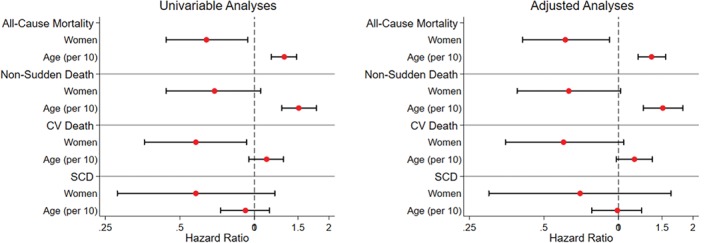
Forest plots demonstrating unadjusted and adjusted hazard ratios for the primary and secondary endpoints stratified by sex and age. CV, cardiovascular; SCD, sudden cardiac death.

All‐cause mortality [hazard ratio (HR) 0.64, 95% confidence interval (CI) 0.44–0.94; P = 0.020] and cardiovascular death (HR 0.58, 95% CI 0.36–0.93; P = 0.025) were lower in women compared to men with similar trends for non‐sudden (HR 0.68, 95% CI 0.44–1.05; P = 0.088) and sudden death (HR 0.58, 95% CI 0.28‐1.21; P = 0.15) (Figure 2 and Table 4 ). Following adjustment for age, LVEF, NYHA class, AF, LBBB, smoking status, LGE and CRT or ICD implantation, all cause‐mortality (HR 0.61, 95% CI 0.41–0.92; P = 0.018) was lower in women compared to men with similar trends for cardiovascular (HR 0.60, 95% CI 0.35–1.05; P = 0.07) and non‐sudden death (HR 0.63, 95% CI 0.39–1.02; P = 0.06) (Figure 2 and Table 4). Trends were similar in patients with LVEF <40% and those with LVEF ≥40% (online supplementary Table S1). During follow‐up, of those with a LVEF ≤35% at baseline, 32 (32.3%) women and 99 (38.8%) men underwent ICD implantation (P = 0.27). Of those with a LVEF ≤35% and LBBB at baseline, 37 (74.0%) women and 43 (59.7%) men received CRT (P = 0.12). Women with LBBB had lower mortality compared to men with LBBB (HR 0.39, 95% CI 0.20–0.78; P = 0.008). This was not significantly different from the HR for women without LBBB compared to men without LBBB (HR 0.81, 95% CI 0.52–1.26; P = 0.35; heterogeneity P = 0.086). Of those with an ICD, the rate of appropriate shocks was similar for women and men (HR 0.94, 95% CI 0.47–1.89; P = 0.86). Of those without an ICD, women tended to be less prone to sudden death than men but this did not achieve statistical significance (HR 0.60, 95% CI 0.29–1.27; P = 0.18).

**Table 4:** Univariable and multivariable analyses for the primary and secondary endpoints

	**All‐cause mortality**		**Cardiovascular death**		**Non‐sudden death**		**Sudden cardiac death**	
	**HR (95% CI)**	***P*‐value**	**HR (95% CI)**	***P*‐value**	**HR (95% CI)**	***P*‐value**	**HR (95% CI)**	***P*‐value**
HR for women vs. men								
Univariable	0.64 (0.44–0.94)	0.020	0.58 (0.36–0.93)	0.025	0.68 (0.44–1.05)	0.088	0.58 (0.28–1.21)	0.15
Multivariable*	0.61 (0.41–0.92)	0.018	0.60 (0.35–1.05)	0.074	0.63 (0.39–1.02)	0.060	0.70 (0.30–1.63)	0.41
HR based on age (per 10 years increase)								
Univariable	1.32 (1.17–1.48)	<0.00001	1.12 (0.95–1.31)	0.18	1.51 (1.29–1.78)	<0.00001	0.92 (0.73–1.15)	0.47
Multivariable†	1.36 (1.20–1.55)	<0.0001	1.16 (0.98–1.37)	0.078	1.51 (1.26–1.82)	<0.00001	0.99 (0.78–1.24)	0.90

CI, confidence interval; HR, hazard ratio.

* Adjusted for left ventricular ejection fraction, New York Heart Association class, atrial fibrillation, left bundle branch block, smoking, the presence of late gadolinium enhancement, age and the presence of an implantable cardioverter‐defibrillator or cardiac resynchronisation therapy as time varying covariates.

† Adjusted for left ventricular ejection fraction, New York Heart Association class, atrial fibrillation, left bundle branch block, smoking, the presence of late gadolinium enhancement, sex and the presence of an implantable cardioverter‐defibrillator or cardiac resynchronisation therapy as time varying covariates.

### Association between age and outcome

All‐cause mortality increased with age (per 10 years: HR 1.32, 95% CI 1.17–1.48; *P* < 0.00001), largely driven by a rise in the rate of death from non‐sudden causes (per 10 years: HR 1.51, 95% CI 1.29–1.78; *P* < 0.00001) (*Figure* 2 and *Table* 4). Death from cardiovascular (HR 1.12, 95% CI 0.95–1.31; *P* = 0.18) and sudden causes (per 10 years: HR 0.92, 95% CI 0.73–1.15; *P* = 0.47) did not significantly change with advancing age. Results were similar in univariable and multivariable analyses (*Figure* 2 and *Table* 4). Trends were similar in patients with LVEF <40% and those with LVEF ≥40% (online supplementary *Table* S1). During follow‐up, of those with a LVEF ≤35% at baseline, 86 (39.1%) of those <60 years of age and 45 (36.3%) of those older underwent ICD implantation (*P* = 0.91). Of those with a LVEF ≤35% and LBBB at baseline, 46 (70.8%) of those <60 years of age underwent CRT compared to 34 (59.6%) of those older (*P* = 0.25). Of those with an ICD, there was no difference in the rate of appropriate shocks with advancing age (per 10 years: HR 0.89, 95% CI 0.71–1.11; *P* = 0.30). Of those without an ICD, there was no difference in the rate of sudden death between those aged >60 years compared to younger patients (per 10 years: HR 0.90, 95% CI 0.72–1.12; *P* = 0.35).

**Figure 3:**
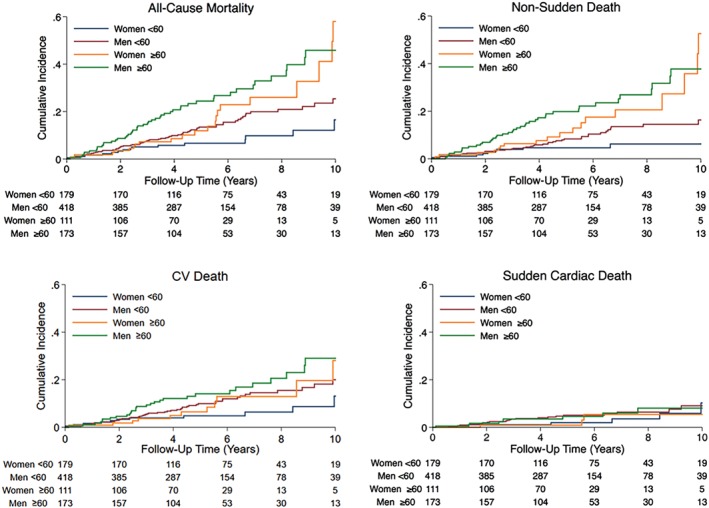
Cumulative incidence curves demonstrating the occurrence of endpoints based on the age and sex of patients. CV, cardiovascular.

In keeping with the proportional hazard analysis, cumulative incidence curves demonstrated increased all‐cause mortality in patients over 60 years of age compared to those younger that was driven by death from non‐sudden causes, without a similar rise in sudden death (*Figure* 3). The rise in all‐cause mortality and non‐sudden death was less marked in women compared to men. In women under the age of 60 years, 5‐year mortality estimates from Kaplan–Meier curves was 6.7% (95% CI 3.7–11.8) compared to 11.9% (95% CI 6.7–21.0) in those older. In men under the age of 60 years, 5‐year mortality estimates from Kaplan–Meier curves was 13.5% (95% CI 10.3–17.5) compared to 24.4% (95% CI 18.3–32.2) in those older.

## Discussion

This is the first study to specifically examine the impact of sex and age on the phenotype and outcome of DCM in a well characterised population. Outcomes with contemporary treatment were favourable. Overall, women had lower mortality than men even after adjusting for several key prognostic variables, including implanted devices. For example, women under the age of 60 years had an estimated 5‐year mortality rate of only 6.7% compared to 13.5% in men under 60 years of age. Sudden death accounted for only 27.5% of overall mortality, in keeping with recent data from Shen and colleagues demonstrating a reduction in this mode of death with current HF therapy.^16^ The slightly higher rate of CRT amongst women, reflecting the higher prevalence of LBBB, did not account for the sex differences in outcome. Interestingly, women had more severe symptoms despite having less severe cardiac dysfunction, lower burden of scar and similar pharmacological therapy compared to men. Our data also show that mortality is higher in patients over 60 years of age and that this is predominantly driven by death from non‐sudden causes rather than sudden death.

A detailed description of differences in the outcome of men and women in a broad well characterised DCM population has been lacking until now. For patients with HF of mixed aetiology, several studies have reported a lower mortality amongst women compared to men but this may reflect the higher prevalence of coronary artery disease amongst men, which carries a worse prognosis.^3^ Studies in patients with DCM secondary to specific genetic mutations also suggest that men have a worse prognosis than women, however, it has been unclear whether this is genotype‐specific or more general.^17^, ^18^ Our study in patients with well characterised DCM is not confounded by coronary artery disease or specific to small subgroups with specific genetic causes.

This study offers several possible explanations for the better prognosis amongst women with DCM including less severe cardiac dysfunction and lower scar burden. Similar to previous multicentre registries there was a predominance of men in our study, making up almost 70% of the cohort.^4^ A greater susceptibility to developing ventricular impairment in men may explain this disparity. Truncating mutations in titin are thought to make individuals susceptible to developing contractile impairment and men with such variants have been shown to have worse outcome than women.^18^ Protection from cardiovascular disease in pre‐menopausal women has been linked with sex hormones, including estradiol.^19^ In patients with arrhythmogenic cardiomyopathy estradiol appears to have a protective and testosterone a detrimental effect across both sexes.^20^ Increased levels of estradiol reduced myocyte apoptosis in an *in vitro* model of arrhythmogenic cardiomyopathy, while increased testosterone levels potentiated it. Myocyte death is central to the development of replacement fibrosis and it is possible that the different impact of these sex hormones on myocyte survival contributes to a higher prevalence of replacement fibrosis in men. A sex disparity in the prevalence of replacement fibrosis in DCM is consistent with other studies and has also been demonstrated in acute myocarditis and aortic stenosis.^21^, ^22^, ^23^ Other studies have demonstrated sex differences in gene expression in patients presenting with HF secondary to DCM and these may be responsible for differences in phenotype and outcome.^24^

In our cohort, a greater percentage of women were referred following a presentation with HF whilst an arrhythmic presentation was more common in men. In keeping with this and similar to previous studies in patients with HF, women reported more severe functional limitation compared to men.^2^ Whether the greater HF symptom burden in women is explained by differences in pathophysiology, symptom reporting or perception is unclear. HF secondary to diastolic dysfunction is more common in women^3^ but in our cohort, LAVi, a useful marker of chronically elevated filling pressure, was smaller in women. Other markers of diastolic function, exercise performance and natriuretic peptides were not available for the current analysis but would provide interesting insights.

Left bundle branch block was more common in women compared to men. This observation is particularly interesting as LBBB is often attributed to more advanced disease, however, in our study women had other markers of less severe disease. Previous work in patients receiving CRT demonstrated that LBBB is associated with better survival in women compared to men, even when controlling for co‐morbidities.^25^ Our data also demonstrated greater survival in women with LBBB compared to men with LBBB, despite similar rates of CRT. The mechanism explaining the greater incidence of LBBB in women and whether the prognostic significance of LBBB differs between sexes merits further research.

Our study also suggests that caution should be exercised with regard to the implantation of ICDs in patients over 60 years of age due to an increased risk of death from competing causes, lending support to the DANISH trial that demonstrated an absence of overall survival benefit with ICD therapy in patients aged >59 years and to data from previous clinical trials.^5^, ^8^ Our data confirm that the lack of survival benefit with ICD therapy in older patients is because a high proportion of deaths are non‐sudden rather than a lower risk of arrhythmic death.

### Limitations

This cohort, although large, was enrolled in a single centre and has a relatively low prevalence of common co‐morbidities such as diabetes mellitus. It is possible that this reflects a degree of selection bias; however, our referral base is broad, from specialist and non‐specialist centres and the baseline characteristics are similar to other cohorts.^26^ The referral characteristics and specifically the proportion of men and women referred remain stable over the study period (online supplementary *Figure* S1). This approach also enables detailed CMR phenotyping using well established protocols generating a well characterised population.

For some secondary endpoints, we had fewer events, limiting statistical power. Differences in disease characteristics between men and women may reflect differences in the time taken to seek medical attention after the onset of symptoms. However, the difference in all‐cause mortality persisted following adjustment for indicators of disease severity at referral. Information on sex‐specific variables including obstetric history, use of hormone replacement therapy or an oral contraceptive, age of menopause and previous gynaecological surgery was not available.

We also recognise that a proportion of sudden deaths are likely to relate to non‐cardiac events such as aneurysmal rupture and cerebral haemorrhage. In the absence of routine autopsy data, assuming a cardiac aetiology to all sudden deaths could result in over‐estimation of the overall incidence of sudden cardiac death. Nevertheless, in keeping with recent data, the incidence of sudden death in our study was low.^16^

## Conclusion

The prognosis of women with DCM is, on average, better than for men. This may be partly attributed to a disease course characterised by less severe ventricular dysfunction and a smaller scar burden. The chance of death due to causes other than arrhythmias increases with age, rendering ICDs less effective in reducing all‐cause mortality. Our data emphasise the importance of developing sex‐ and age‐specific risk stratification and management approaches.

### Funding

B.P.H. is supported by a British Heart Foundation Clinical Research Training Fellowship (FS/15/29/31492). V.S.V. has received grant support from Rosetrees Charity Trust. J.G.F.C. has received non‐financial research support from Boston Scientific and Medtronic. D.J.P. has received research support from Siemens and is a stockholder and director of CVIS. S.K.P. has received grant support from the British Heart Foundation, the Alexander Jansons Foundation and the Rosetrees Charity Trust.

**Conflict of interest:** none declared.

## Supporting information


**Table S1.** Univariable and multivariable analyses for the primary and secondary endpoints.
**Figure S1.** Proportion of referral population that were women based on year of baseline scan.
