# Supplementary material for: Sex‐ and age‐based differences in the natural history and outcome of dilated cardiomyopathy
Source: Eur J Heart Fail. 2018 Jun 3;20(10):1392–400. doi: 10.1002/ejhf.1216 (PMC6392171; doi:10.1002/ejhf.1216)
Supplement: Supplementary file 1 — Table S1. Univariable and multivariable analyses for the primary and secondary endpoints. Figure S1. Proportion of referral population that were women based on year of baseline scan. [file EJHF-20-1392-s001.doc]

**SUPPLEMENTARY MATERIAL**

**Table S1 Univariable and multivariable analyses for the primary and secondary endpoints**

**a. Subgroup LVEF <40% (**n=436)

| **HR for Women vs Men** | **All-Cause Mortality** | | **Cardiovascular Death** | | **Non-Sudden Death** | | **SCD** | |
| --- | --- | --- | --- | --- | --- | --- | --- | --- |
|  | **HR (95% CI)** | **P** | **HR (95% CI)** | **P** | **HR (95% CI)** | **P** | **HR (95% CI)** | **P** |
| Univariable | 0.70 (0.45, 1.08) | 0.11 | 0.66 (0.48, 1.14) | 0.13 | 0.72 (0.44, 1.18) | 0.19 | 0.67 (0.25, 1.80) | 0.43 |
| Multivariable* | 0.62 (0.38, 1.01) | 0.053 | 0.63 (0.33, 1.22) | 0.17 | 0.66 (0.38, 1.15) | 0.15 | 0.70 (0.22, 2.17) | 0.53 |
| **HR based on Age**  **(per 10 years increase)** | **All-Cause Mortality** | | **Cardiovascular Death** | | **Non-Sudden Death** | | **SCD** | |
|  | **HR (95% CI)** | **P** | **HR (95% CI)** | **P** | **HR (95% CI)** | **P** | **HR (95% CI)** | **P** |
| Univariable | 1.20 (1.04, 1.38) | 0.015 | 1.02 (0.85, 1.23) | 0.82 | 1.31 (1.08, 1.57) | 0.005 | 0.86 (0.62, 1.19) | 0.35 |
| Multivariable† | 1.35 (1.16, 1.58) | <0.001 | 1.15 (0.93, 1.40) | 0.15 | 1.38 (1.13, 1.68) | 0.001 | 0.97 (0.70, 1.35) | 0.86 |

**b. Subgroup LVEF ≥40% (**n=445)

| **HR for Women vs Men** | **All-Cause Mortality** | | **Cardiovascular Death** | | **Non-Sudden Death** | | **SCD** | |
| --- | --- | --- | --- | --- | --- | --- | --- | --- |
|  | **HR (95% CI)** | **P** | **HR (95% CI)** | **P** | **HR (95% CI)** | **P** | **HR (95% CI)** | **P** |
| Univariable | 0.60 (0.29, 1.22) | 0.16 | 0.48 (0.18, 1.28) | 0.15 | 0.73 (0.29, 1.85) | 0.50 | 0.49 (0.17, 1.47) | 0.21 |
| Multivariable* | 0.56 (0.26, 1.23) | 0.15 | 0.59 (0.19, 1.85) | 0.36 | 0.46 (0.18, 1.17) | 0.10 | 0.83 (0.23, 2.97) | 0.77 |
| **HR based on Age**  **(per 10 years increase)** | **All-Cause Mortality** | | **Cardiovascular Death** | | **Non-Sudden Death** | | **SCD** | |
|  | **HR (95% CI)** | **P** | **HR (95% CI)** | **P** | **HR (95% CI)** | **P** | **HR (95% CI)** | **P** |
| Univariable | 1.43 (1.15, 1.78) | 0.001 | 1.15 (0.84, 1.58) | 0.38 | 2.06 (1.42, 2.97) | <0.001 | 0.98 (0.71, 1.35) | 0.89 |
| Multivariable† | 1.44 (1.13, 1.84) | 0.003 | 1.16 (0.82, 1.63) | 0.41 | 2.05 (1.32, 3.17) | 0.001 | 0.97 (0.69, 1.38) | 0.88 |

*adjusted for left ventricular ejection fraction, New York Heart Association Class, atrial fibrillation, left bundle branch block, smoking, the presence of late gadolinium enhancement, age and the presence of an implantable cardioverter defibrillator or cardiac resynchronisation therapy as time varying co-variates

† adjusted for left ventricular ejection fraction, New York Heart Association Class, atrial fibrillation, left bundle branch block, smoking, the presence of late gadolinium enhancement, sex and the presence of an implantable cardioverter defibrillator or cardiac resynchronisation therapy as time varying co-variates

(ASCD – aborted sudden cardiac death, HF – heart failure, HR – hazard ratio, SCD – sudden cardiac death)

**Figure S1 Proportion of referral population that were women based on year of bsaeline scan**

**
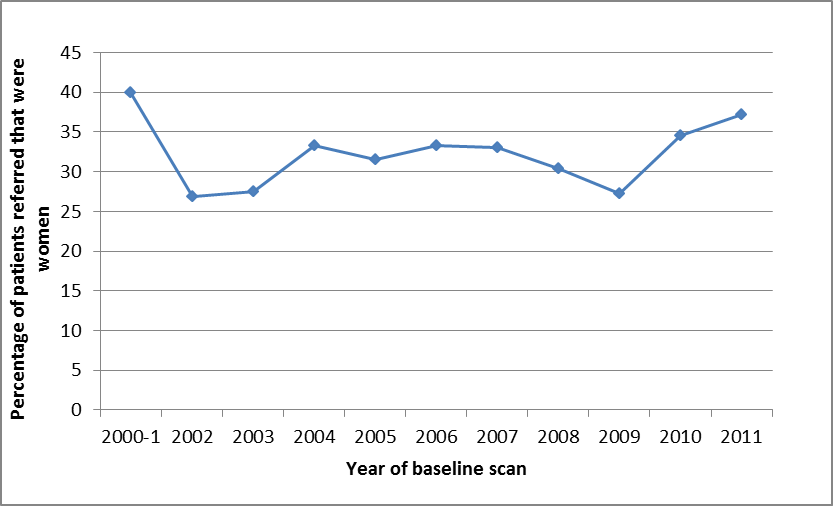
**
